# The implication of protein malnutrition on cardiovascular control systems in rats

**DOI:** 10.3389/fphys.2015.00246

**Published:** 2015-09-02

**Authors:** Fernanda C. Silva, Rodrigo C. de Menezes, Deoclécio A. Chianca

**Affiliations:** ^1^Laboratory of Cardiovascular Physiology, Department of Biological Sciences, Institute of Biological Sciences, Federal University of Ouro PretoOuro Preto, Brazil; ^2^Graduate Program in Biological Sciences – CBIOL/NUPEB, Federal University of Ouro PretoOuro Preto, Brazil

**Keywords:** protein malnutrition, neuroplasticity, sympathetic activity, cardiovascular reflexes, renin-angiotensin system

## Abstract

The malnutrition in early life is associated with metabolic changes and cardiovascular impairment in adulthood. Deficient protein intake-mediated hypertension has been observed in clinical and experimental studies. In rats, protein malnutrition also increases the blood pressure and enhances heart rate and sympathetic activity. In this review, we discuss the effects of post-weaning protein malnutrition on the resting mean arterial pressure and heart rate and their variabilities, cardiovascular reflexes sensitivity, cardiac autonomic balance, sympathetic and renin-angiotensin activities and neural plasticity during adult life. These insights reveal an interesting prospect on the autonomic modulation underlying the cardiovascular imbalance and provide relevant information on preventing cardiovascular diseases.

## Introduction

Malnutrition, an important pathological condition resulting from deficient intake or absorption of macro and/or micronutrients, reaches more than 900 million individuals worldwide and accounts for about 3.5 million deaths of under 5 year-old children (Black et al., 2008). Clinical and experimental researches propose that malnutrition in early life stages is often associated with metabolic and cardiovascular disorders in adult life (Plagemann et al., 2000; Langley-Evans, 2007). From a hypothetical “nutritional programming” point of view, the nutritional deficiency can prompt epigenetic alterations, including the compromising of the autonomic nervous system (ANS), which gives rise to secondary metabolic and cardiovascular disturbances, such as: insulin resistance, coronary disease, and hypertension (Benabe and Martinez-Maldonado, 1993; Lucas, 1998; Barker, 2007). It is not a novelty that low protein intake impairs the cardiovascular homeostasis, increasing the mean arterial pressure (MAP), which is an important cardiovascular risk factor (Handler and Bernheim, 1950; Engen and Swenson, 1969). However, the pathophysiological mechanisms underlying it remain under investigation. The understanding of the cardiovascular changes caused by protein malnutrition comprises two points: understanding the mechanisms that control blood pressure (BP) in this nutritional state, and looking for changes that precede alterations on BP and other variables that influence the cardiovascular homeostasis.

Cardiovascular control involves feedback systems activation, which operates on short and long terms (Shepherd and Mancia, 1986; Dampney, 1994). The short term regulation mechanisms comprise the cardiovascular reflexes. In this regard, peripheral information detected by specific receptors is processed in the central nervous system (CNS) and returns to the periphery by efferent ANS subdivisions: sympathetic nervous system (SNS) and parasympathetic nervous system, to maintain homeostasis (Machado et al., 1997). The long term regulation mechanisms relate to humoral systems, such as renin-angiotensin system (RAS) whose unbalance contributes to the development/maintenance of high peripheral resistance and vascular hyper-reactivity observed in hypertension (Ferguson and Bains, 1997; Mendoza and Lazartigues, 2015). Both, short- and long-term regulation systems play important roles in physiologic and pathologic conditions. The pathogenesis of cardiovascular diseases (e.g., heart failure, coronary disease, and hypertension) can be associated with unbalanced autonomic cardiovascular regulation, particularly by SNS overactivation (Sinski et al., 2006). Although it has been speculated that sympathetic hyperactivity could be the key alteration, the chronological sequence between impaired sympathetic drive and other abnormalities have not yet been determined (Mancia and Grassi, 2014). Regarding of malnutrition-mediated autonomic cardiovascular disorders, it is essential to identify the mechanisms that are underlying to this process for setting cause-consequence relationship. In this sense, we will discuss the following aspects in the present review: (a) resting MAP and heart rate (HR) and their variabilities, (b) cardiovascular reflexes sensitivity, (c) SNS activity/reactivity, (d) RAS activity, and (e) neural plasticity related to dysfunctional cardiovascular autonomic control observed in the experimental protein malnutrition model.

## Post-weaning protein malnutrition experimental model

Our laboratory has been focused to studying the cardiovascular disorders observed in adult rats submitted to post-weaning protein malnutrition protocol. This model reflects a situation that occurs in underdeveloped countries, where the newborn receives a satisfactory amount of protein by breast feeding, only to see the protein intake reduced after weaning (Rodrigues et al., 2012).

In gestation and weaning periods, females receive regular rat chow and filtered water *ad libitum*. After the weaning period (28 days), male rats are separated from their mothers and kept in grouped cages. Over the next 35 days, rats are fed either a normal or a low protein diet, which make up our two experimental groups: normal protein (NP) and low-protein (LP), respectively. The regular diet contains 15% protein while the low protein diet contained 6%. The diets are isocaloric (422 kcal/100 g of diet) and the salts and vitamins are also similar in both, as described in Table 1. Animals are submitted to experiments from 36th up to 41st day (Tropia et al., 2001; Oliveira et al., 2004; Loss et al., 2007; Penitente et al., 2007; Bezerra et al., 2011a,b; Martins et al., 2011a; Rodrigues et al., 2012; Rodrigues-Barbosa et al., 2012; Gomide et al., 2013; Silva et al., 2013).

**Table 1:** **Composition (g/100 g of diet) of both control and low protein diets (Tropia et al., 2001; Bezerra et al., 2011b)**.

**Components**	**NP group**	**LP group**
Casein	15	6
Cornstarch	70	79
Soy oil	8	8
Mineral mixture	5	5
Vitamin mixture	1	1
Fiber (Cellulose)	1	1
Energy density, kcal	422	422

In order to attest the efficiency of our dietary restriction protocol we measured: body weight, hematocrit, total blood protein, plasma albumin, urea, glucose, and food ingestion in both groups (Tropia et al., 2001; Oliveira et al., 2004; Penitente et al., 2007). The LP group presented lower values in all parameters above mentioned, except for hematocrit percentage, urea concentration and daily food ingestion, which were similar compared to NP group.

## The influence of post-weaning low-protein diet on resting MAP and HR and their variabilities

Clinical and experimental studies have being showing, for over five decades, that low protein intake could lead to blood pressure increase, which is one of the most important cardiovascular risk factors (Handler and Bernheim, 1950; Viart, 1978; Sawaya et al., 2005; Martins et al., 2011b; Barros et al., 2015; de Brito Alves et al., 2015). Furthermore, data from our group indicated that cardiovascular reflexes are impaired by protein dietary restriction, see below (Tropia et al., 2001; Loss et al., 2007; Penitente et al., 2007; Bezerra et al., 2011a). Since cardiovascular reflexes are required to modulate MAP and HR (Morais et al., 2015), defective cardiovascular reflex sensitivity is correlated to abnormal variability of these parameters (Oliveira et al., 2004). Studies have also indicated that the risk to cardiovascular complications may depend on BP increase, as well as changes in MAP and HR variabilities (Shaffer et al., 2014; Parati et al., 2015).

MAP variability (MAPV) increase, on short-, mid- or long-term, could predict the development, progression and severity of cardiovascular injury and mortality (Parati et al., 2015). Likewise, HR variability (HRV), which reflects autonomic modulation of the heart (Task Force of European Society of Cardiology, 1996), has been used to identify autonomic changes in pathophysiological cardiovascular complications (e.g., hypertension and heart failure) (Souza et al., 2008; Shaffer et al., 2014). Therefore, in this section, we will discuss the influence of post-weaning low-protein diet on resting MAP and HR and their variabilities.

Low protein diet increases resting MAP and HR, accompanied by an increased variability of both parameters in rats (Oliveira et al., 2004). Among possible mechanisms involved, two stand out: elevated sympathetic activity and/or increased action of vasoactive substances (e.g., angiotensin), which will be better approached in following topics of this review. In this work, the application of advanced acquisition methodology along with more precise analysis tools concurred to better highlight differences in resting HR and MAP, as expected in cases of elevated sympathetic and RAS activities (Shaffer et al., 2014; Parati et al., 2015). Higher MAPV and HRV observed in malnourished rats were predictable once the gain feedback control loops were also raised (Oliveira et al., 2004). Consequently, little MAP variations may result in HR over corrections due to enhanced cardiovascular reflexes gain feedback mechanisms (Tropia et al., 2001; Penitente et al., 2007; Bezerra et al., 2011a), which is in accordance with our data proposing an enhanced sympathetic tonus in rats submitted to protein deficient dietary (Tropia et al., 2001).

## The influence of post-weaning low-protein diet on cardiovascular reflexes sensitivity

### Baroreflex

Baroreflex provides moment-to-moment negative feedback regulation of BP. Carotid sinus and aortic baroreceptors distension generates electrical signals, which are transmitted to the nucleus tractus solitary (NTS), where the first baroreflex synapse, probably using a glutamate as the neurotransmitter, occurs (Talman et al., 1980). Projections from NTS stimulate caudal ventrolateral medulla (CVLM) neurons, which in turn inhibit the rostral ventrolateral medulla (RVLM). Thereby, central sympathetic outflow is suppressed, since RVLM neurons send projections to preganglionic neurons in the intermediolateral column, which comprise the sympathoexcitatory output to the periphery (Schreihofer and Guyenet, 2003; Kumagai et al., 2012). Briefly, a prompt BP increase, which activates baroreflex, enhances the cardiovagal activity and reduces the cardiac and vascular sympathetic activity. This lessens the HR and corrects the BP to appropriate levels. On the order hand, a BP decrease deactivates baroreflex. Therefore, cardiovagal activity is suppressed while cardiac and vascular sympathetic activity is amplified, causing HR elevation and BP adjustment (Vasquez et al., 2012).

The baroreflex control is altered in rats fed a low-protein diet. Protein malnutrition increases the baroreflex activation latency and bradycardia gain evoked by phenylephrine (PHE), without changing the baroreflex deactivation latency and sodium nitroprusside-mediated tachycardia gain (Tropia et al., 2001; Loss et al., 2007).

Regarding the efferent autonomic activity influence on the baroreflex activation in LP rats, the latency was further enhanced and the bradycardic gain remained increased after methyl-atropine intravenous (i.v.) administration. However, after metoprolol i.v. injection, the latency was not affected, but the bradycardic gain decreased (Loss et al., 2007). These data suggest an increased sympathoinhibition and a decreased parasympathetic excitation during baroreflex activation in rats that had a low protein intake.

Considering the baroreflex deactivation in LP rats, the latency increased and the tachycardia gain decreased after methyl-atropine i.v. injection, suggesting an impairment in sympathetic activity modulation. In contrast, after metoprolol injection, these parameters were not affected (Loss et al., 2007). In accordance to another models of malnutrition studies (Herlihy et al., 1992; VanNess et al., 1997; de Belchior et al., 2012), these data indicate that low-protein diet disrupts cardiovascular regulation driven by baroreflex loops.

### Chemoreflex

Chemoreflex, another important cardiovascular reflex, comprises peripheral, and central chemoreceptors, mainly located in carotid bodies and brainstem, respectively. The first one is activated mainly by hypoxia while the second one is stimulated fundamentally by hypercapnia (Nurse, 2010; Mansukhani et al., 2014). In this topic, we considered the carotid body chemoreceptor contribution on autonomic control. The carotid body afferents send projections to NTS neurons, which project to RVLM and nucleus ambiguous, respectively, controlling sympathetic and parasympathetic outflows (Schreihofer and Guyenet, 2003; Kumagai et al., 2012; Accorsi-Mendonça and Machado, 2013). Chemoreflex, whose activation stimulates breathing and sympatho/parasympathoexcitatory efferent pathways resulting in pressor and bradycardic responses (Barros et al., 2002), is involved in systemic hypertension onset, since its chronic activity could trigger a sustained MAP rise (Fletcher et al., 2002). In this sense, studies have been performed to address whether the protein malnutrition disrupts the cardiovascular autonomic control driven by chemoreflex pathway.

The carotid body artery ligature, which degenerates chemosensitive cells resulting in impairment of chemoreflex activation, further enhanced baseline MAP and HR in LP rats (Penitente et al., 2007). Previous report, in which carotid body artery ligature reduced baseline MAP in normotensive rats, pointed out the inhibitory effect of chemoreflex response on baroreflex (Franchini and Krieger, 1992). In fact, other results showed that peripheral chemoreflex activation attenuates baroreflex activation (Gu et al., 2007; Yamamoto et al., 2013). However, in our data, carotid body chemoreflex seems to exert stimulatory effect on baroreflex during protein malnutrition, suggesting that such nutritional condition reversed these mechanisms. Thus, specific changes in the central interplay of baroreflex and chemoreflex could justify our findings (Penitente et al., 2007). The reduced parasympathetic and enhanced sympathetic efferent modulation, repeatedly observed in malnourished rats (Benabe and Martinez-Maldonado, 1993; Plagemann et al., 2000; Tropia et al., 2001) also support such hypothesis.

This data is unique by pointing a BP increase as a consequence of chemoreflex response removal in post-weaning protein malnourished rats (Penitente et al., 2007). In this study, PHE-mediated baroreflex activation or baroreflex deactivation by sodium nitroprusside (SNP) i.v. injection produced the expected cardiovascular responses. So, MAP and HR detected changes in ligated-LP rats were not evoked by afferent baroreflex denervation, as a ligature surgery artifact (Penitente et al., 2007). Chemoreflex activation, by potassium cyanide (KCN) i.v. injections, elicits dose-related pressor and bradycardic responses, which are abolished by carotid body arteries ligature. All tested KCN doses (5, 10, 15, 20, and 40 μg/kg) produced higher decrease in HR, while just the three smaller doses elicited greater pressor responses in malnourished rats, suggesting that low-protein diet increases basal efferent sympathetic tonus (Penitente et al., 2007). Such increased sympathetic activity mediated by malnutrition could be a consequence of following mechanisms: reduction in nitric oxide synthesis (Efron and Barbul, 1999), increases in angiotensin plasma and tissue levels, enhance in angiotensin II mRNA expression (Benabe and Martinez-Maldonado, 1993; Benabe et al., 1993a,b; Tonkiss et al., 1998), as well as enhances in chemoreflex activation and/or peripheral/central chemosensitive responses (Agarwal et al., 1981; de Brito Alves et al., 2015). Supporting the last hypothesis, the hypoxia-inducible factor expression, a transcriptional factor which relates to hypoxia-mediated tissue response and energy availabilities, was enhanced in rats submitted to another protein restriction model, suggesting a sensitization of the carotid peripheral chemoreceptors (de Brito Alves et al., 2015). Moreover, a recent work showed that carotid body chemoreflex activation-mediated pressor response remained elevated even in recovered protein restricted rats (Sá et al., 2014). In view that the chemoreflex pressor response depends on sympathetic activation (Vieira et al., 2012), they proposed that the sympathoexcitation arising from chemoreflex stimulation could remain enhanced in these rats (Sá et al., 2014).

The aforementioned pointed that protein malnutrition enhances cardiovascular responses to carotid chemoreflex activation in conscious rats. As this autonomic imbalance seems to alter the interplay between baroreflex and chemoreflex neuronal mechanisms, it could be considered a risk factor and could set deleterious effects on cardiovascular homeostasis (Penitente et al., 2007).

### Bezold–Jarish reflex

The autonomic control of the circulation also depends on the cardiopulmonary reflexes (Verberne and Guyenet, 1992). The Bezold–Jarisch reflex evoked by unmyelinated cardiopulmonary fibers (C fibers) activation, is characterized by hypotension, bradycardia, and apnea (Thorén, 1979). The C fibers arise from receptors located in the atria, ventricles, aorta and lungs, traveling through the vagus nerve up to the NTS (Kalia and Mesulam, 1980). In fact, besides the central integrative areas, BJR and baroreflex share afferent and efferent cardiovascular pathways, interplaying in a inhibitory manner (Verberne and Guyenet, 1992; Kashihara, 2009). In this sense, in the absence of arterial baroreceptors, the Bezold–Jarisch reflex plays an essential role in the reflex control of circulation, since its responsiveness is enhanced in this condition (Chianca et al., 1997). Considering that post-weaning protein malnutrition disrupts baroreflex and chemoreflex regulation, studies were performed to evaluate its influence on the Bezold-Jarisch reflex.

In a study from our laboratory, Bezold–Jarisch reflex activation, by serotonin injection, induced dose dependent hypotension and bradycardia in NP and LP rats. But, hypotension in LP was higher than in NP in the maximal dose used, whereas bradycardia was greater in all doses tested (Tropia et al., 2001). In a latter study Bezold–Jarisch reflex was activated by phenylbiguanide (5-HT3 serotonin receptor agonist) in NP and LP rats before and after baroreflex denervation (Bezerra et al., 2011a). In this study, protein restriction did not affect the Bezold-Jarisch reflex responses (hypotension and bradycardia). Nevertheless, after baroreflex denervation, such cardiovascular responses were attenuated in malnourished rats. It displayed a reduced BJR responsiveness in LP after baroreceptors removal (Bezerra et al., 2011a).

In conscious rats, hypotension evoked by Bezold–Jarisch reflex activation depends on bradycardia, indicating a plausible role of parasympathetic drive for Bezold–Jarisch reflex-mediated cardiovascular response (Chianca et al., 1997). The relationship between hypotension and bradycardia, evaluated by ΔMAP/ΔHR index, were increased only in animals fed a low protein diet submitted to baroreflex denervation, suggesting that Bezold–Jarisch reflex-evoked sympathetic and parasympathetic responses are, in some way, dissociated in malnourished rats without baroreflex (Bezerra et al., 2011a). In order to verify if that was a specific condition caused by protein malnutrition in baroreflex absence, Bezold–Jarisch reflex was evaluated along with muscarinic blockade in intact NP and LP rats. Our results showed similar pattern response to Bezold–Jarish reflex activation for both groups and proposed that a higher ΔMAP/ΔHR ratio observed in denervated malnourished rats was closely related to the absence of the afferent baroreceptor signals to CNS (Bezerra et al., 2011a). Since the inhibition of baroreflex medullary pathways declines Bezold–Jarisch reflex responsiveness associated with a parallel baroreflex blockade/attenuation (Verberne and Guyenet, 1992), we speculated that the protein malnutrition may result in Bezold–Jarisch reflex higher dependency on the baroreflex at CNS level, so that the absence of the last could lessen the efficacy of the first (Bezerra et al., 2011a). Although more investigations are required for better understanding of this phenomenon, these results strongly indicate that low protein diet changed the interrelation between Bezold–Jarisch reflex and baroreflex required for BP maintenance (Bezerra et al., 2011a).

## The influence of post-weaning low-protein diet on sympathetic activity/reactivity

The initial assessment of heart autonomic activity were preformed using the intrinsic HR and HRV methods (Task Force of European Society of Cardiology, 1996). The data pointed out that low protein diet increases the sympathetic and decreased the parasympathetic tone. Parasympathetic blockade, by a muscarinic receptor antagonist (methylatropine) i.v. injection, increased resting HR in animals fed a normal diet, but not in malnourished rats. This indicates that protein malnutrition could reduce the vagal modulation to HR (Martins et al., 2011a). Sympathetic blockade, by a selective β1-adrenoceptor antagonist (metoprolol) i.v. injection, reduces HR in animals fed a low protein diet but has no effect on animals under a normal diet. This data strongly suggests that a low-protein diet increases the sympathetic efferent activity to the heart (Martins et al., 2011a). In fact, recent finding corroborates this data showing that protein malnutrition increased sympathetic activity in rats (Barros et al., 2015; de Brito Alves et al., 2015). The double injection of methylatropine and metoprolol reduces HR in malnourished rats, but again has no effect on animals control animals, indicating that malnourished rats have a low intrinsic HR (Martins et al., 2011a). Intrinsic HR can be modified by changes in the centrally mediated sympathetic and/or vagal flow. In addition, intrinsic HR has important role on resting HR providing an important compensatory mechanism to maintain HR in appropriated levels during an autonomic activity unbalance. In this sense, an increase in sympathetic activity along with a decrease in vagal activity leads to intrinsic HR reduction (Machado and Brody, 1989).

Moreover, the cardiac autonomic index, used to measure the sympathetic and parasympathetic activity balance (Goldberger, 1999), was < 1 in rats fed a low protein diet, indicating sympathetic dominance in these rats. Interestingly, this index was higher in control than in malnourished rats, suggesting parasympathetic activity dominance in the former (Martins et al., 2011a). This study also demonstrated that the LF/HF ratio was increased in malnourished rats when compared to control rats, in accordance to a recent study, which performed HRV analysis in a different malnutrition model (Barros et al., 2015). These results also pointed to the greater sympathetic activity effect than parasympathetic activity on cardiac autonomic balance in protein malnutrition (Martins et al., 2011a).

Several mechanisms can reflect such autonomic unbalance, including alterations in the synthesis/release of neurotransmitters (Penido et al., 2012) and morphological damage in CNS circuitry recruited in the genesis and/or modulation of autonomic activity (Plagemann et al., 2000; Pinos et al., 2011). It is well established that chronic cardiac sympathetic activation raises the sudden death risk (Schultz and Li, 2007; Pokorný et al., 2011) and malnutrition could trigger cardiovascular disturbances (Sawaya et al., 2005), highlighting the fundamental importance of keeping investigate the autonomic balance in protein-deprived situation.

Among the strong evidence that malnutrition mediates sympathetic overdrive our group evaluated the impact of post-weaning protein malnutrition on the SNS activity. Blockade of α1-adrenoreceptor in malnourished rats caused greater depressor and tachycardia responses when compared to animals fed a regular diet, suggesting an increased vascular sympathetic tone (Tropia et al., 2001). Indeed, a recent work showed that another post-weaning protein malnutrition model, along with BP raise, induces vascular dysfunction, revealed by increases in superoxide anion, nitric oxide, and vascular reactivity of resistance arteries (de Belchior et al., 2012).

Faced with all findings previously discussed, our group considered necessary to assess the SNS responsiveness to malnutrition by a direct methodology. So, we measured renal sympathetic reactivity, directly, during Bezold-Jarisch reflex stimulation in rats submitted to post-weaning protein restriction (Bezerra et al., 2011b). The Bezold-Jarisch reflex activation besides producing hypotension, bradycardia and apnea, reduces renal sympathetic nerve activity in order to exert the homeostatic control of blood volume (Ditting et al., 2006). Although studies showed that Bezold–Jarisch reflex differentially regulates sympathetic drive to different regions, the Bezold–Jarisch reflex activation plays a specific control on renal nerve (Veelken et al., 1993).

Bolus i.v. injection of phenylbiguanide (5 μg/kg) evoked transient drops in renal sympathetic nerve activity of NP and LP rats. However, renal sympathetic reactivity was substantially diminished in malnourished rats (Bezerra et al., 2011b). In this context, considering the ability of renal nerves regulate blood volume and vascular resistance, renal sympathetic overdrive can contribute to the development and progression of cardiovascular disorders (Barrett, 2015). Since we observed similar renal sympathetic nerve activity- mediated MAP and HR drops in NP and LP rats (Bezerra et al., 2011a), changes in peripheral serotonin receptors expression and their effectiveness, as previous described (Chen et al., 1992), could be discarded. However, concerning that low-protein diet modified the cardiovascular reflexes responsiveness (Tropia et al., 2001; Loss et al., 2007; Penitente et al., 2007; Bezerra et al., 2011a,b) and their central pathway have some common medullary structures (Kashihara, 2009), we hypothesized that protein malnutrition may also impairs brain mediated autonomic control, which will be better discussed in the last topic of this review.

## The influence of post-weaning low-protein diet on renin-angiotesin system activity

Circulating and local renin-angiotensin system (RAS) components are strictly associated with cardiovascular complications, especially in the development and progression of hypertension. Angiotensin II (Ang II), the main effector of this system, exerts its action on specific receptor isoforms, AT1 and AT2. When Ang II binds the AT1 receptor, it prompts vasoconstriction, cell proliferation, and hypertension (Santos and Ferreira, 2007; Mendoza and Lazartigues, 2015).

Studies have shown that low protein dietary enhances RAS activity, contributing to BP levels increase (Martinez-Maldonado et al., 1993; Benabe et al., 1993a; Goyal et al., 2010). Indeed, protein deprivation in early life increases mRNA expression to numerous RAS components in many tissues (Sangaleti et al., 2004; Goyal et al., 2009). As a result, an interaction between ANS and RAS activation might produce cardiovascular adaptations detected in adult rats submitted to post-weaning protein restriction.

A recent study from our group showed that an interaction between Ang II and the SNS contributes to the BP increase observed in rats fed a low-protein diet (Gomide et al., 2013). Although previous studies have emphasized the specific influence of RAS or SNS on cardiovascular regulation in experimental low-protein dietary condition (Martinez-Maldonado et al., 1993; Martins et al., 2011a), our results are the first to exhibit the interaction between increased RAS and SNS drive as accountable for the BP maintenance in protein malnutrition (Gomide et al., 2013).

Enalapril injection decreases the MAP in LP rats, but does not alter the basal MAP in NP rats. Moreover, such reduction reached lower MAP levels than that observed in NP group (Gomide et al., 2013). This indicates that RAS is important for the small MAP elevation also previously observed in malnourished animals (Oliveira et al., 2004; Loss et al., 2007; Penitente et al., 2007), as well as an essential regulatory mechanism, which could prevent a potential chronically hypotensive state in these animals (Gomide et al., 2013).

Since RAS seems to be involved in the BP regulation in protein-restricted rats, we also conducted experiments to evaluate the role of AT1 receptors on the their BP. Losartan injection also decreased the MAP in LP rats (Gomide et al., 2013), in accordance to previous reports in which losartan by gavage administration during 5 days reduced MAP in rats fed a low-protein diet (Benabe et al., 1993b). Our data revealed that Ang II, acting in AT1 receptors, is an essential factor for the BP maintenance in rats undergoing protein restriction. Interestingly, LP rats presented much lower levels of circulating Ang II than NP rats (Gomide et al., 2013). These data support early results demonstrating that protein restriction reduced both Ang II plasma concentration and plasma renin activity (Fernández-Repollet et al., 1987; Kapoor and Krishna, 1991).

Moreover, increasing doses of Ang II produced smaller MAP raises in LP than NP rats. Given that the basal RAS activity is enhanced in LP rats, it is plausible to suppose that the AT1 receptors are saturated in these animals and, therefore, the crescent Ang II doses administered produced less pronounced effects in LP than in NP group (Gomide et al., 2013). The relation between both poor responsiveness to Ang II and its circulating lower levels is an indicative that the RAS adaptation, pointed as one of the main regulatory system accountable for the high BP observed in rats fed a low-protein diet, is not due to an increase in the Ang II plasma levels, but due to a likely AT1 receptors overexpression in arteries and/or in the CNS (Gomide et al., 2013). In fact, we found increased AT1 receptors expression in the aorta of LP rats. It is important to note that, in the NP group, we did not observe any significant changes in the MAP or HR after enalapril or losartan injections alone (Gomide et al., 2013). These findings are in tune with previous data showing that, with an adequate content-protein diet, the RAS plays no major role in the moment-to-moment maintenance of BP and HR (Ceravolo et al., 2007).

In order to better understand the mechanisms underlying BP control in rats fed a low-protein diet, we evaluated the relative contribution and the possible interaction between RAS and SNS on the BP regulation in these animals. Under AT1 receptor blockade, prazosin infusion further reduced MAP in LP rats, suggesting that Ang II, acting on AT1 receptors, could activate SNS resulting in BP raise (Gomide et al., 2013). It is known that these two systems display positive feedback interplay in CNS and vasculature, in which raised activity of one of them increases the output of the other (Mancia et al., 2006). In addition to this data that shows an increased RAS activity in LP rats, previous data from our laboratory have indicated that these animals also present a higher vasomotor tone probably due to an increased sympathetic efferent activity (Tropia et al., 2001).

As expected, when prazosin was injected before losartan, MAP decreased in both groups. Prazosin-mediated MAP reduction in LP group was analogous to the losartan effect, also indicating the strong AT1 receptors contribution to the BP maintenance in protein restriction condition (Gomide et al., 2013). The following losartan injection further decreased MAP in LP rats. In NP group, prazosin injected alone also reduced MAP. However, such reduction was smaller than in LP rats, confirming that an increased sympathetic drive is required to sustain the raised BP levels after protein restriction. Moreover, both drugs injection, regardless of the order, reduced MAP in NP group, although these responses were smaller than in LP rats (Gomide et al., 2013). This information also converges to the understanding that protein-restricted rats need a higher AT1 receptor activity to maintain an appropriate sympathetic tone to the vascular bed.

To the point, our results displayed that in protein restriction condition, the α1-receptors activation is under strong influence of Ang II acting on AT1 receptors, demonstrating that Ang II is crucial to support the vascular tone driven by the SNS in this situation. RAS (specifically Ang II) and the SNS are both hyperactived, contributing in a complementary manner to maintain the BP levels in LP in order to preserve the cardiovascular system, and maintain sufficient blood supply to the systems. Therefore, the interplay between the RAS and the SNS appears to occur, in the arteries since AT1 receptors expression in the aorta is higher in LP rats (Gomide et al., 2013). However, more investigations are required to reveal if this interaction occurs mainly in the periphery, as suggested by the increased AT1 expression, or whether it is also a consequence of the CNS activation (e.g., by circumventricular organs), which probably increase the sympathetic drive.

## The influence of post-weaning low-protein diet on neural plasticity

Neural plasticity, an adaptive process which changes the CNS structure and function during any ontogeny stage, is a result of internal/external environment interactions, or even of neural injuries (Phelps, 1990). According to literature, malnutrition, during critical development periods, reduces the quantity and span of dendritic processes, decreases the synapse/neuron relation (Nordborg, 1978; Díaz-Cintra et al., 1990; Morgane et al., 2002; Cordero et al., 2003; Penido et al., 2012), decreases the thickness myelin sheath and internodal segments (Reddy et al., 1979; Quirk et al., 1995; Cordero et al., 2003), impairs the release and activity of glutamate (Rotta et al., 2003; Penido et al., 2012) and produces morphophysiology alterations in brain regions which are involved in cardiovascular control, such as hypothalamus, hippocampus, frontal cortex, and amygdala (Plagemann et al., 2000; Zhang et al., 2009; Flores et al., 2011; Matos et al., 2011; Pinos et al., 2011). Such neural adaptations could change the electrical conduction system and modify the cardiac autonomic outflow, as was proposed by a recent study, in which cardiovascular responses induced by central injection of α-type scorpion toxin were attenuated in protein restricted rats (Silva et al., 2013).

These observations support our idea that cardiovascular impairment observed in post-weaning malnutrition experimental model might be related to CNS plasticity. In this review, we have already presented data that pointed to a cardiac autonomic dysfunction as a protein malnutrition consequence (Tropia et al., 2001; Oliveira et al., 2004; Loss et al., 2007; Penitente et al., 2007; Bezerra et al., 2011a,b; Martins et al., 2011a; Gomide et al., 2013). In this regard, the impairment of cardiovascular reflexes observed in experimental protein malnutrition, may be triggered by any central plasticity whose magnitude is able to interfere in cardiovascular homeostasis.

Would protein malnutrition be able to change the specific brain nuclei responsiveness to intermittent baroreflex stimulation? In order to investigate this hypothesis, we assessed the expression of neuronal activity marker c-fos protein (immediate-early gene expression) in the paraventricular hypothalamus (PVH); NTS; rostral ventromedial medullary areas (RVMM); raphe pallidus (RPa) and raphe obscurus (Rob); caudal ventrolateral medullary areas (CVLM) and RVLM (Rodrigues-Barbosa et al., 2012).

Baroreflex intermittent activation modifies c-fos expression in the PVH, RPa, medial NTS, and CVLM, independently on the dietary protocol offered to rats. However, in response to baroreflex stimulation, protein restricted dietary protocol influenced the neuronal recruitment pattern in raphe obscurus and in important medullary nuclei of cardiovascular control (rostral and caudal-commissural NTS, RVMM, and RVLM) (Rodrigues-Barbosa et al., 2012).

It is known that RVMM neurons conduct the sympathetic drive to heart and thermogenesis (Cao and Morrison, 2003; Salo et al., 2006; Morrison and Nakamura, 2011; Morrison et al., 2012). Additionally, RVMM neurons activation mediates marked tachycardia (Cao and Morrison, 2003, 2006; Morrison, 2003). Phenylephrine infusion induced neuronal activation within the RVMM of rats fed a low protein diet, but not in the control group, denoting that protein restriction is able to change neuronal recruitment, increasing the resting HR to maintain the cardiac output homeostasis. We also showed a lessened RVLM neuronal activation in LP PHE-infused rats (Rodrigues et al., 2012). The sympathoinhibition triggered by baroreflex activation, essential to preserve the cardiac functionally, results from RVLM neuronal inhibition by CVLM GABAergic input (Guyenet, 2006). Although the CVLM c-fos expression has been similar in NP and LP PHE-infused rats, suggesting comparable recruitment of these nuclei, we did not perform any neurotransmission assay in this region. Therefore, in malnutrition condition, the CVLM GABAergic neurotransmission to RVLM could be impaired and/or RVLM neurons could answer in a different manner to CVLM inhibitory inputs (Rodrigues-Barbosa et al., 2012).

PHE-activated baroreflex also showed differential neural recruitment in NTS of NP and LP rats. In medial NTS, Phe infusions similarly enhanced c-fos expression in both groups, while in rostral and caudal-commissural NTS this expression were higher in LP than NP rats (Rodrigues-Barbosa et al., 2012). These observations support the idea that protein restricted diet could promote differential neural setting in the NTS, a nucleus recognized for receiving and processing afferent cardiovascular information (Guyenet, 2006; Accorsi-Mendonça and Machado, 2013).

Facing to these findings, new assessment of neurochemical plasticity in medullary neurons could become a powerful strategy to deeply understand the autonomic and cardiovascular effects evoked by protein malnutrition.

Based on scarcity of studies about the malnutrition effect on CNS nuclei of cardiovascular control and assuming that: (i) RVLM is an important area for the generation of sympathetic efferent drive, especially to vasomotor tone (Guyenet, 2006; Fontes et al., 2011) and (ii) L-glutamate is the principal excitatory neurotransmitter in this nucleus (Machado et al., 1997), our group also investigated the impact of protein restriction on L-glutamate-mediated pressor response into RVLM (Rodrigues et al., 2012).

Crescent doses of L-glutamate injection into the RVLM evoked smaller pressor responses in LP than NP rats (Rodrigues et al., 2012). Allied to this, protein malnutrition lessened and shifted upward the baroreflex curve, once again indicating the malfunctioning of this reflex in LP rats (Rodrigues et al., 2012). The range of baroreflex gain, which was higher in LP than in NP rats, was in accordance to maximum baroreflex gain. While this occurred at normal levels in NP rats, in LP rats the peak occurred at higher values. In addition, despite of the higher resting HR, the baroreflex activation, by pressor response to glutamate injection in RVLM, produced smaller HR changes in LP when compared to NP rats (Rodrigues et al., 2012). This is another indication that protein malnourished rats present sympathetic efferent overdrive.

All aforementioned observations could be partially explained by damage in glutamate release and/or receptor affinity mediated by protein malnutrition. This would be in concordance to prior studies in the literature which reported that malnutrition, during the first life stages, changes the neurotransmitter concentration in CNS, the neurotransmitter/receptor affinity, the neuronal population, and/or CNS nucleus morphology (Plagemann et al., 2000; Zippel et al., 2003). Although we cannot assure any morphological plasticity, which would characterize and emphasize the changes in the structure of synapses and neurons, our findings point to glutamate neurochemical plasticity. Therefore, post-weaning malnutrition indeed impacts the central mechanisms related to cardiovascular control, particularly considering the glutamate neurotransmission in RVLM—a crucial brainstem nucleus accountable for modulating the sympathetic drive to the cardiovascular system (Rodrigues et al., 2012).

## Conclusions

**Figure 1:**
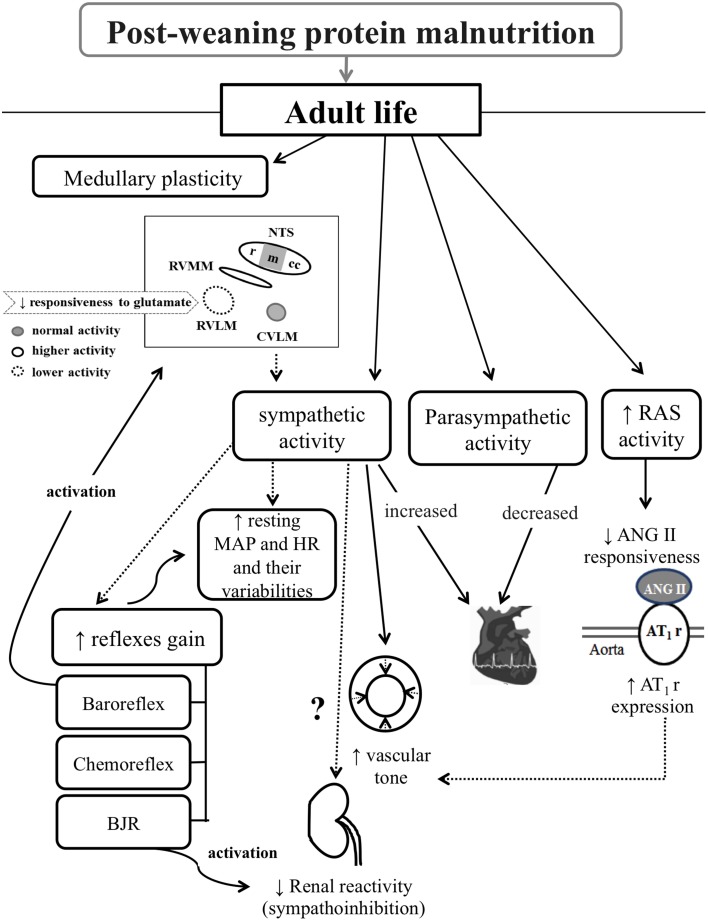
**Diagram illustrating the post-weaning protein malnutrition effects on cardiovascular control systems in adult rats**. Rectangles and black arrows correspond to data observed in this malnutrition model. Dashed arrows correspond to likely interaction between observed data. From left to right, protein restriction promotes: medullary plasticity, vasomotor and cardiac sympathetic overactivity, decreased cardiac parasympathetic activity, and RAS hyperactivity. The neuroplasticity is characterized by changes in medullary recruitment in response to baroreflex stimulation, and decreased glutamate responsiveness in the RVLM. Such medullary plasticity probably contributes to the sympathetic overactivity. This, in turn, relates to the impaired cardiovascular reflexes sensitivity, increased resting MAP and HR and their variabilities, and plausible shift in baseline renal sympathetic activity in view of the reduced renal sympathoinhibition to BJR activation. The RAS hyperactivity associates with the low ANG II responsiveness and high aortic AT_1_r expression, which probably contribute to increased vascular tone. RAS, renin-angiotensin system; MAP, mean arterial pressure; HR, heart rate; BJR, Bezold–Jarish reflex; ANG II, angiotensin II; AT_1_r, angiontensin II receptor type 1; NTS, nucleus tractus solitary; rNTS, rostral NTS; mNTS, medial NTS; ccNTS, caudal-commissural NTS; RVMM, rostral ventromedial medullary areas; RVLM, rostral ventrolateral medullary area; and CVLM, caudal ventrolateral medullary area.

This review aimed at pointing out the protein malnutrition impact on cardiovascular homeostasis, since it: (i) impairs the cardiovascular reflexes sensitivity, (ii) increases resting MAP and HR and their variabilities, (iii) enhances the sympathetic and diminishes the parasympathetic efferent activities to the heart, (iv) raises the vasomotor sympathetic tonus, (v) reduces the renal sympathoinhibition to BJR activation, (vi) increases the RAS activity, and (vii) changes the medullary recruitment and glutamate neuromodulation in response to baroreflex stimulation, as outlined in Figure 1.

Thus, the present review provides new perspectives on the pathophysiology of metabolic and cardiovascular diseases associated with protein malnutrition.

### Conflict of interest statement

The authors declare that the research was conducted in the absence of any commercial or financial relationships that could be construed as a potential conflict of interest.
